# Progressive Medicine, Vol. III, September, 1907

**Published:** 1907-11

**Authors:** 


### BOOK REVIEWS.

PROGRESSIVE MEDICINE, VOL. III. SEPTEMBER, 1907. A Quarterly Digest of Advances, Discoveries and Improvements in the Medical and Surgical Sciences. Editer by Hobart Amory Hare, M.D., Professor of Therapeutics and Materia Medica in the Jefferson Medical College of Philadelphia. Octavo, 290 pages, with 15 engravings. Per annum, in four cloth-bound volumes, $9.00; in paper binding, $6.00, carriage paid to any address. Lea Brothers & Co., Publishers, Philadelphia and New York.

This volume is made up of four major articles, each of which aims at condensing in itself all the recent work pertaining to its subject, and gives us in an hour the digested information that it would otherwise take months to acquire. In each the wise selection and arrangement brings out forcibly, to eye and mind, what might actually be obscured by a wider and more diffuse view.

Dr. William Ewart's article on "Diseases of the Thorax and Its Viscera, Including the Heart, Lungs and Blood-vessels," contains an epitome of recent work on Tuberculosis, valuable especially since the whole subject is so unsettled—the views of the workers so diversified, and some of their discoveries so radical in effect, if true, that not to keep up with them is to lose one's hold on modern medical life. Here the progress of medicine seems almost a retrogression when we compare the views of Koch with the conclusions of the Royal Commission in its investigation into the relations of bovine and human tuberculosis, or Dr. Nathan Rau with his antagonism between the two types, and Weber, who differs from him in part and agrees in part. The conclusion of the author (P. 19,) when he says, "If bovine infection is in reality answerable for all tuberculosis other than pulmonary, then a large proportion of this scourge as it now prevails could be stopped by an efficient and absolute control over milk, butter, cheese and all dairy products," and, following out this path, blind though it be, the author advances the novel idea that if these two varieties of tubercle be antagonistic, then in ridding ourselves of the danger from one, we should, in the opinion of one investigator, be depriving ourselves of our main method of immunity and cure.

But that pulmonary tuberculosis is due to inhalation of bacilli is by no means an accepted view, as a perusal of Dr.

Ewart's paper and its references to recent work will speedily convince the reader. The method of conveyance of extraneous matter—bacilli—or mineral or vegetable substances is being studied with a view to settling this very point, and here again we find conflicts between the views and observations of Calmetti and those who, with equal zeal, oppose him, each backed up with experimental evidence, leaving it still unsettled whether tuberculosis is air-borne or infested, or both.

Dermatology and Syphilis, by William S. Gottheil, M.D., begins with a list of don'ts that prove to be mostly do's, but are just as useful. The author remarks on the great change that has taken place in the last year in the attitude of the X-ray students in dermatology. The tendency, he says, is to limit the sphere of the X-rays more and more, and to realize its perils. Considerable space is devoted to Trypsine. Some of the minor subjects of the article are Dermatitis Vegetans—Endothelioma of the Skin—Feigned Eruptions—the hair in disease—the diagnostic and prognostic value of Herpes, in which last subject the reader will, we think, find much that is novel. Affections of the nails gives us another very useful topic, and in the section on syhpilis all the new work will be found. The prophylactic use of mercury locally is strongly advocated by the author. The article has many illustrations.

Obstetrics by Dr. Edward P. Davis, and Diseases of the Nervous System, by Wm. G. Spiller, M.D., conclude the volume.

